# Response of 5 hpf zebrafish embryos to low-dose microbeam protons

**DOI:** 10.1093/jrr/rrt164

**Published:** 2014-03

**Authors:** Viann Wing Yan Choi, Candy Yuen Ping Ng, Alisa Kobayashi, Teruaki Konishi, Masakazu Oikawa, Shuk Han Cheng, Peter Kwan Ngok Yu

**Affiliations:** 1 Department of Physics and Materials Science, City University of Hong Kong, Hong Kong; 2 Department of Technical Support and Development, National Institute of Radiological Sciences, 4-9-1 Anagawa, Inage, Chiba 263-8555, Japan; 3 Department of Biology and Chemistry, City University of Hong Kong, Hong Kong; 4 State Key Laboratory in Marine Pollution, City University of Hong Kong, Hong Kong

**Keywords:** protons, dose–response, zebrafish embryos

## Abstract

The microbeam irradiation system (single-particle irradiation system to cell, acronym as SPICE) at the National Institute of Radiological Sciences (NIRS), Japan, was employed to irradiate dechorionated embryos of the zebrafish, *Danio rerio*, at 5 h post-fertilization (hpf) by protons each having an energy of ∼3.4 MeV. Either 1 or 10 positions on the cells were irradiated with different number of protons. The levels of apoptosis in zebrafish embryos at 25 hpf were quantified through terminal dUTP transferase-mediated nick end-labeling assay. Triphasic dose–esponses were obtained (Fig. 1), including (i) a subhormetic zone with an increase in apoptotic signals for a small number of irradiated protons per position, (ii) a hormetic zone with a reduction in the apoptotic signals below the spontaneous level for a larger number of irradiated protons per position and (iii) a toxic zone with an increase in apoptotic signals again if the number of irradiated protons per position was further increased.

**Fig. 1.**
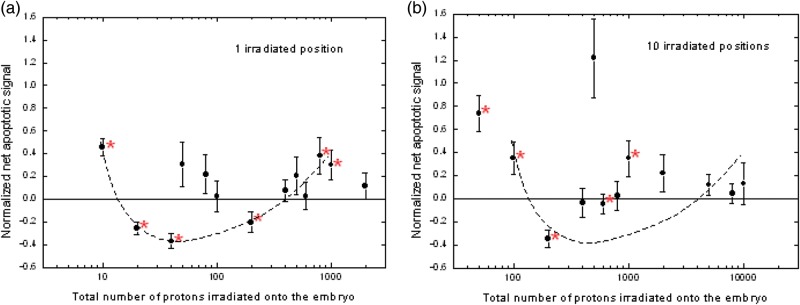
Relationship between normalized net apoptotic signals on 25 hpf zebrafish embryos and the total number of protons irradiated onto the zebrafish embryos at 5 hpf. Asterisked points: significant differences cf. control samples (*t*-tests: *P*≤ 0.05). (**a**) Data for irradiation at 1 position. Broken line: trend shown by asterisked points. (**b**) Data for irradiation at 10 positions. Broken line: same as the one shown in (a) for comparison.

Relationship between normalized net apoptotic signals on 25 hpf zebrafish embryos and the total number of protons irradiated onto the zebrafish embryos at 5 hpf. Asterisked points: significant differences cf. control samples (*t*-tests: *P*≤ 0.05). (**a**) Data for irradiation at 1 position. Broken line: trend shown by asterisked points. (**b**) Data for irradiation at 10 positions. Broken line: same as the one shown in (a) for comparison.

Clinical Trial Registration number if required: None

